# The Association of Dietary Behaviors and Physical Activity Levels with General and Central Obesity among ASEAN University Students

**DOI:** 10.3934/publichealth.2017.3.301

**Published:** 2017-06-23

**Authors:** Karl Peltzer, Supa Pengpid

**Affiliations:** 1 HIV/AIDS/STIs and TB (HAST) Research Programme, Human Sciences Research Council, Private Bag X41, Pretoria 0001, South Africa; 2 Department of Research and Innovation, University of Limpopo, Sovenga 0727, South Africa; 3 ASEAN Institute for Health Development, Mahidol University, Salaya, Phutthamonthon, Nakhonpathom 73170, Thailand

**Keywords:** general obesity, central obesity, dietary behavior, physical activity, sedentary behavior, university students, ASEAN

## Abstract

**Objective:**

To quantify the prevalence of obesity and obesity-related factors (dietary behaviors and physical activity levels) in a cross-sectional, observational study of ASEAN undergraduate students.

**Material and Methods:**

A total of 6783 (35.5% male and 64.5% female) undergraduate students (Mean age: 20.5, SD = 2.0) from eight ASEAN countries completed questionnaires and anthropometric measurements. Multivariable logistic regression was used to estimate odds ratios (ORs) for the association of nutrition behaviors with prevalence of general obesity (body mass index ≥ 25 kg/m^2^), elevated waist-to-height ratio (WHtR) (>0.50), and high waist circumference (WC) (≥80 cm in females, ≥90 cm in males). Covariates included sociodemographic factors, dietary behavior, physical activity and sitting time (using the “International Physical Activity Questionnaire”).

**Results:**

There was a higher prevalence of general obesity (24.2% versus 9.3%), and high WHtR (16.6% versus 12.1) in males relative to females, while high WC (9.4% versus 10.4%) did not significantly differ between genders. In multivariable logistic regression analyses, compared to females, males had higher odds of obesity (odds-ratio, OR: 2.13, confidence interval, CI: 1.80, 2.77), and high WHtR (OR: 1.90, CI: 1.48, 2.43) (*P* < 0.001 for both). Snacking frequency and avoiding fatty foods were associated with all three obesity indicators; obesity (OR: 1.16, CI: 1.05, 1.28 and OR: 1.54, CI: 1.24, 1.92, respectively), WHtR (OR: 1.17, CI: 1.04, 1.32 and OR: 1.46, CI: 1.04, 1.54), and high WC (OR: 1.16, CI: 2.01, 1.33 and OR 1.52, CI: 1.14, 2.04, respectively). Physical activity and sedentary behavior were not significantly associated with any obesity measure.

**Conclusions:**

There was a low prevalence of healthy behaviors and a high prevalence of obesity in this sample of ASEAN young adults. Specific dietary behaviors but not physical activity nor sedentary behavior were associated with obesity.

## 1. Introduction

Globally, the prevalence of obesity has more than doubled from 1980 to 39% of adults (≥18 years) being overweight and 13% being obese in 2014 [1]. In the Association of Southeast Asian Nations (ASEAN) region, the adult (>20 years) prevalence of overweight (BMI ≥ 25–29.99 kg^2^) and obesity (BMI ≥ 30 kg^2^) ranged from in Vietnam (13.6% and 1.5%, respectively, among men, and 12.3% and 1.7%, respectively, among women) to in Malaysia (43.8% and 11.4% among men, and 48.6% and 16.7% among women) [2]. Globally and in the Southeast Asian region, obesogenic behaviors such as the consumption of “energy-dense foods that are high in fat, salt and sugars but low in vitamins, minerals and other micronutrients” and physical inactivity and sedentary behavior have increased, contributing to overweight and obesity, including in young adults [3]. Obesity is a major risk factor for a number of non-communicable diseases, such as “diabetes mellitus, cardiovascular disease, hypertension and stroke, and certain forms of cancer” leading to the “increased risk of premature death to serious chronic conditions that reduce the overall quality of life” [4]. It is important to understand factors driving the obesity epidemic in ASEAN so as to better design health interventions [5].

Emerging adulthood is an important period in health behavior development as many of the health behaviors may change due to changing influences of parents, peers, social contexts and identity development [6]–[8]. Health risk behaviors such as dietary behavior, physical activity and sedentary behavior and the prevalence of obesity in university students has not been well studied in ASEAN [9]–[12]. Therefore, the aim of this study was to quantify the prevalence of obesity and obesity-related factors (dietary behaviors and physical activity levels) in a cross-sectional study of ASEAN undergraduate students.

## 2. Materials and Methods

### 2.1. Sample and procedure

This was a cross-sectional study using a self-administered questionnaire and anthropometric measurements in university students in eight ASEAN countries. The questionnaire utilized for data collection was developed in English, then translated and back translated into the languages (Bahasa, Lao, Myanmar, Thai, and Vietnamese) of the participating countries. In every participating study country, the questionnaire was pre-tested on a sample of 20–30 university students for validity; these results did not form part of the final sample. Some of the instruments used in this study have previously been validated, such as the International Physical Activity Questionnaire (IPAQ). In each study country, undergraduate students were surveyed in classrooms (inclusion criteria were present on the day of the survey) selected through a cluster random sample procedure (one university department randomly selected from each faculty as a primary sampling unit, and for each selected department randomly ordered undergraduate courses). Informed consent was obtained from participating students, and the study was conducted from 2014 to 2015. Participation rates were in all countries more than 90%, except for Indonesia 69% and Myanmar 73%. Ethics approvals for the study protocol were obtained from all participating institutions.

### 2.2. Measures

*Anthropometric measurements.* Students' anthropometric measurements were recorded by trained researchers using standardized protocols [13]. Standing height was measured to the nearest 0.1 cm without shoes, using a stadiometer. Participants wearing light clothes, were weighed to the nearest 0.01 kg, on a load-cell-operated digital scale which was first calibrated using a standard weight and re-checked daily [14]. Waist circumference was measured around the waist through a point one third of the distance between the xiphoidprocess and the umbilicus, using a non-stretchable tape measure [15]. Body mass index (BMI) was calculated as weight in kg divided by height in meters squared. BMI was classified according to Asian criteria: normal weight (18.5 to <23.0), overweight (23.0 to <25.0) and 25+ as obese [16]. Central obesity was defined as Waist Circumference (WC) 90 cm or more in men and 80 cm or more in women [17],[18]. Waist-to-height ratio (WHtR) was defined as their waist circumference divided by their height, both measured in the same units; elevated WHtR was defined as >0.5 [19].

Socio-demographic questions included age, gender and year of study. Socioeconomic background was assessed by relative family income within each country as wealthy (within the highest 25%), quite well off (within the 50% to 75% range), not very well off (within the 25% to 50% range), or quite poor (within the lowest 25%); students were classified as wealthy if they considered themselves as wealthy or quite well off and poor if they considered themselves as coming from a not very well off or quite poor family background [20].

*Dietary behavior variables:* Fruit and vegetable (FV) consumption was assessed with two questions “How many servings of fruit do you eat on a typical day?” and “How many servings of vegetables do you eat on a typical day?” (One standard serving = 80 g) [21]. Cronbach alpha for this fruit and vegetable measure was 0.76. Additional dietary variables included: (a) frequency of consumption of red meat (daily, 2–3 times a week, once a week, less than once a week, never); (b) trying to avoid eating foods that contain fat and cholesterol (yes, no); (c) trying to eat foods that are high in fiber (yes, no); (d) frequency of having breakfast; (e) frequency of between-meal snacks and (f) number of meals a day [20].

*Physical activity* was assessed using the self-administered International Physical Activity Questionnaire (IPAQ) short version, for the last 7 days (IPAQ-S7S). We used the instructions given in the IPAQ manual for reliability and validity, which is detailed elsewhere [22]. We categorized physical activity (short form) according to the official IPAQ scoring protocol [23] as low, moderate and high.

*Sitting time* was measured with the IPAQ short form [23]: *“During the last 7 days, how much time did you usually spend sitting on a weekday? Include time spent at work, at home, while doing course work and during leisure time. This may include time spent sitting at a desk, visiting friends, reading,* *or sitting or lying down to watch television”* [23]. The question was answered in terms of hours and minutes. The average sitting time per day in minutes was calculated, and 480 minutes (8 hours) were classified as high sitting time or sedentary behavior [24].

### 2.3. Data analysis

The data were analyzed using IBM SPSS Statistics for Windows (Version 20.0. Armonk, NY: IBM Corp, USA). Sociodemographic factors, dietary variables, physical activity levels, and obesity categories were calculated as percentages. Multivariable logistic regression was performed with three obesity indicators (BMI obesity, high WC and high WHtR) as the dependent variables. Potential multi-collinearity between variables was assessed with variance inflation factors, none of which exceeded (1.8) critical value. Socio-demographic characteristics, dietary variables, physical activity levels and sedentary behavior were taken as independent variables. *P* < 0.05 was considered significant. The country was entered as the primary sampling unit for survey analysis in STATA in order to achieve accurate CIs, given the clustered nature of the data.

## 3. Results

### 3.1. Sample characteristics

The sample included 6783 (35.5% male and 64.5% female) university students (Mean age: 20.5, SD = 2.0) from eight ASEAN countries. The sample size differed by country, ranging from 333 in Myanmar to 1583 in Thailand. Classifying students into wealthy and poor family background, most (62.4%) considered themselves as poor, especially in Laos (95.3%) and Vietnam (96.6%). Overall, the number of students was more or less equally distributed across the study years from 1 to 4 or 5, while students from Laos and Myanmar had higher proportions of students in higher years of study (see Table 1).

### 3.2. Descriptives of dietary behavior, physical activity levels and anthropometric variables

Overall, the consumption of meals were on average 2.7 per day, the number of between meal snacks were on average 1.6, the mean number of fruit servings per day 1.4 and the mean number of vegetable servings per day 1.8. More than half (53.2%) of students did not have regularly their breakfast (not almost every day), 58.7% had at least once a day red meat, 40.2% avoided eating foods high in fat and cholesterol and 48.4% reported trying to eat foods high in fiber. Half of the students (49.9%) had low physical activity, 33.6% moderate and 16.6% high physical activity. Sedentary behavior (sitting of eight or more hours) was reported by 31.5% of the students. The mean body weight for men was 62.3 kg and for women 51.0 kg, the mean height for men was 168 cm and for women 157 cm and the mean BMI was 22.2 for men and 20.8 for women. The prevalence of BMI overweight among men was 12.3% and among women 8.4%, the prevalence of BMI obesity among men was 24.2% and among women 9.3%, the prevalence of high waist-height ratio was among men16.6% and among women 12.1%, and high waist circumference among men was 9.4% and among women 10.1%. Among the different study countries, BMI obesity was the highest in Malaysia (25.6% for men and 15.8% for women, followed by Myanmar (24.2% for men and 15.4% for women) and Indonesia (23.3% for men and 10.6% for women). The highest prevalence of high waist/height ratio was in Indonesia (30.9% for men and 11.9% for women) and Malaysia (21.4% among men and 20.1% for women) and the Philippines (20.9% among men and 10.6% among women). Similarly, the prevalence of high waist circumference was the highest in Indonesia (23.6% among men and 8.5% among men) and Malaysia (14.5% among men and 17.7% among women) (see Table 2).

**Table 1.** Sample characteristics.

*Variable*	*Indonesia*	*Laos*	*Malaysia*	*Myanmar*	*Philippines*	*Singapore*	*Thailand*	*Vietnam*	*All*
	*N*	*N*	*N*	*N*	*N*	*N*	*N*	*N*	*N*
*Total*	969	759	1022	333	765	677	1583	765	6783
Men	283	260	519	201	194	347	1289	402	2357
Women	686	499	503	132	571	328	294	363	4514
	M (SD)	M (SD)	M (SD)	M (SD)	M (SD)	M (SD)	M (SD)	M (SD)	M (SD)
*Age*	19.4 (2.5)	22.3 (1.8)	20.7 (1.5)	20.1 (1.2)	18.7 (1.0)	21.2 (1.6)	20.0 (1.3)	21.3 (1.7)	20.5 (2.0)
	%	%	%	%	%	%	%	%	%
*Wealthy family background*	37.3	4.7	58.8	29.7	39.9	60.7	27.9	3.4	37.6
*Year of study*									
1	23.4	0.0	37.9	0.0	28.4	29.8	36.9	19.5	27.5
2	22.2	0.0	27.7	21.3	27.5	27.8	16.3	19.2	20.1
3	32.6	63.6	15.1	0.0	26.6	29.0	32.6	19.6	27.6
4 or 5	21.8	36.4	19.4	78.7	17.5	13.4	14.2	41.7	24.9

M = Mean; SD = Standard deviation.

**Table 2.** Dietary behavior, physical activity and anthropometric variables among ASEAN undergraduate students.

*Variable*	*Indonesia*	*Laos*	*Malaysia*	*Myanmar*	*Philippines*	*Singapore*	*Thailand*	*Vietnam*	*All*
	*M (SD)*	*M (SD)*	*M (SD)*	*M (SD)*	*M (SD)*	*M (SD)*	*M (SD)*	*M (SD)*	*M (SD)*
***Behavior***									
*No meals/day*	2.6 (0.7)	2.9 (0.5)	2.7 (0.5)	2.6 (0.7)	2.7 (0.6)	2.9 (0.7)	2.6 (0.7)	2.8 (0.6)	2.7 (0.7)
*No snacks/day*	1.8 (1.1)	1.4 (1.1)	1.4 (1.0)	2.2 (1.0)	1.7 (0.9)	1.2 (1.0)	2.1 (1.1)	1.1 (0.8)	1.6 (1.1)
*No servings of fruits/day*	1.4 (0.8)	1.2 (0.8)	1.0 (0.8)	1.8 (1.1)	1.1 (0.8)	1.3 (0.9)	1.8 (1.0)	1.6 (1.1)	1.4 (0.9)
*No servings of vegetables/day*	1.6 (0.9)	1.5 (0.9)	1.6 (0.9)	1.5 (0.7)	1.3 (1.0)	1.7 (0.9)	2.1 (1.1)	2.7 (1.0)	1.8 (1.0)
	%	%	%	%	%	%	%	%	%
*Not almost daily breakfast*	59.5	54.8	49.5	32.5	46.4	37.5	70.6	49.2	53.2
*Read meat at least once a day*	20.6	42.7	52.7	96.0	64.7	79.6	62.2	70.1	58.7
*Avoiding fatty foods and cholesterol*	46.3	29.5	37.7	43.1	28.6	43.7	49.5	33.7	40.2
*Trying to eat fiber*	70.3	27.8	41.8	3.9	31.9	38.3	62.6	75.5	48.4
*Physical activity*									
*Low*	56.3	49.9	39.9	87.5	41.7	38.7	51.0	33.3	49.9
*Moderate*	34.6	21.6	45.8	12.5	38.6	38.6	31.4	41.2	33.6
*High*	9.1	28.5	14.3	0.0	19.7	19.7	17.6	25.5	16.6
*Sitting 8 hours or more a day*	20.4	31.0	17.5	0.0	57.0	34.5	19.5	52.8	31.5
***Anthropometry^1^***									
*Weight (kg)-Male*	65.4 (14.1)	56.7 (7.7)	66.4 (14.9)	68.9 (12.9)	61.0 (13.9)	66.4 (11.8)	64.5 (11.9)	59.7 (7.8)	62.3 (12.8)
*Weight (kg)-Female*	51.5 (11.5)	50.5 (8.8)	53.5 (11.4)	53.4 (13.5)	49.4 (10.4)	52.4 (8.6)	53.6 (9.7)	48.0 (5.3)	51.0 (9.9)
*Height (cms)-Male*	169.4 (7.6)	163.3 (6.8)	169.9 (6.5)	170.2 (8.8)	166.0 (6.9)	173.6 (6.6)	171.7 (6.2)	167.9 (5.2)	168.9 (7.0)
*Height (cms)-Female*	156.6 (6.2)	155.7 (6.4)	157.0 (6.1)	157.6 (7.2)	154.1 (5.6)	160.9 (5.7)	160.7 (5.5)	155.6 (5.2)	157.2 (6.2)
*Waist (cms)-Male*	77.2 (19.2)	74.7 (5.4)	77.8 (12.1)	67.7 (17.0)	76.1 (10.3)	79.3 (8.7)	78.5 (8.8)	75.0 (7.0)	76.2 (10.7)
*Waist (cms)- Female*	69.7 (10.5)	71.2 (7.1)	71.6 (9.5)	63.5 (9.7)	68.3 (7.4)	67.6 (7.4)	71.0 (8.1)	68.9 (5.7)	69.7 (8.2)
*BMI-Male*	22.7 (4.7)	21.3 (2.8)	22.9 (4.9)	23.5 (7.3)	22.1 (4.6)	22.1 (4.0)	21.4 (3.7)	21.2 (2.5)	22.2 (4.6)
*BMI-Female*	20.9 (4.1)	20.8 (3.6)	21.7 (4.3)	21.5 (5.4)	20.7 (3.5)	20.2 (2.9)	20.7 (3.5)	19.8 (2.0)	20.8 (4.3)
	%	%	%	%	%	%	%	%	%
*BMI overweight-Male*	14.8	12.7	12.5	14.4	14.4	17.1	11.2	14.9	12.3
*BMI overweight-Female*	8.0	8.4	11.2	7.5	10.2	9.8	7.3	5.2	8.4
*BMI obesity-Male*	23.3	11.2	25.6	24.2	19.6	12.2	14.3	7.4	24.2
*BMI obesity-Female*	10.6	10.4	15.8	15.4	8.4	5.8	9.9	1.0	9.3
*High WHtR-Male*	30.9	9.3	21.4	14.3	20.9	17.4	12.3	12.3	16.6
*High WHtR-Female*	11.9	14.5	20.1	5.0	10.6	2.2	11.8	6.1	12.1
*High WC-Male*	23.6	2.8	14.5	8.2	10.7	11.7	7.4	3.9	9.4
*High WC-Female*	8.5	10.9	17.7	6.4	7.5	3.3	13.4	3.6	10.1

**^1^** Waist circumference data were only available for a subset of students from Indonesia (N = 231), Singapore (N = 201), and Thailand (N = 479); M: Mean; SD: Standard Deviation; BMI (Body Mass Index) ≥ 25 kg/m^2^ = obesity; WHtR (Waist-to-height ratio of) >0.5 = High; WC (Waist Circumference high) ≥80 cms in females, ≥90 cms in males.

**Table 3.** Multivariable logistic regression with obesity indicators.

*Variable*	*BMI Overweight (23.0 to <25.0 kgm^2^)*	*BMI Obesity (≥=25 kgm^2^)*	*High Waist-Height Ratio (>0.50)*	*High Waist circumference (≥90 for men and ≥80 for women)*
	*AOR (95% CI)*	*AOR (95% CI)*	*AOR (95% CI)*	*AOR (95% CI)*
***Behavior covariates***				
*Not almost daily breakfast*	1.03 (0.83, 1.28)	1.06 (0.85, 1.57)	0.93 (0.72, 1.21)	1.09 (0.81, 1.47)
*Number of meals/day*	0.78 (0.66, 0.92) **	0.87 (0.73, 1.02)	0.76 (0.62, 0.93) **	0.81 (0.64, 1.01)
*Number of snacks/day*	0.91 (0.82, 1.01)	1.16 (1.05, 1.28) **	1.17 (1.04, 1.32) *	1.16 (1.01, 1.33) *
*Number of servings of fruits/day*	1.06 (0.95, 1.19)	0.94 (0.83, 1.08)	0.99 (0.85, 1.15)	0.95 (0.80, 1.13)
*Number of servings of vegetables/day*	0.85 (0.77, 0.95) **	0.99 (0.88, 1.11)	0.95 (0.83, 1.08)	0.88 (0.75, 1.03)
*Eating red meat at least once a day*	1.07 (0.87, 1.32)	0.78 (0.63, 0.96) *	0.98 (0.77, 1.26)	0.89 (0.67, 1.17)
*Avoiding fatty foods and cholesterol*	0.98 (0.79, 1.22)	1.54 (1.24, 1.92) ***	1.46 (1.08, 1.54) **	1.52 (1.14, 2.04) **
*Trying to eat fiber*	1.10 (0.89, 1.37)	1.18 (0.95, 1.48)	1.24 (0.95, 1.60)	1.27 (0.95, 1.72)
*Physical activity*				
*Low*	1 (Reference)	1 (Reference)	1 (Reference)	1 (Reference)
*Moderate*	1.10 (0.88, 1.39)	1.11 (0.88, 1.41)	1.16 (0.88, 1.54)	1.06 (0.77, 1.44)
*High*	1.11 (0.85, 1.47)	0.99 (0.75, 1.32)	1.27 (0.92, 1.74)	0.84 (0.58, 1.24)
*Sitting 8 hours or more a day*	0.89 (0.71, 1.12)	1.04 (1.05, 1.28)	1.16 (0.90, 1.50)	1.27 (0.95, 1.70)
***Sociodemographics***				
*Male gender (base: female)*	1.70 (1.38, 2.10) ***	2.13 (1.80, 2.77) ***	1.90 (1.48, 2.43) ***	1.23 (0.92, 1.64)
*Year of study*				
*One*	1 (Reference)	1 (Reference)	1 (Reference)	1 (Reference)
*Two*	1.07 (0.79, 1.46)	0.89 (0.64, 1.23)	0.93 (0.63, 0.93)	0.69 (0.43, 1.09)
*Three*	1.02 (0.77, 1.35)	1.19 (0.90, 1.58)	1.14 (0.81, 1.60)	1.02 (0.70, 1.49)
*Four or five*	1.10 (0.83, 1.46)	1.19 (0.89, 1.59)	1.07 (0.76, 1.51)	1.01 (0.69, 1.48)
*Wealthy (base: poor)*	1.07 (0.86, 1.32)	1.29 (1.04, 1.60)*	1.22 (0.94, 1.58)	1.24 (0.92, 1.66)

AOR: Adjusted Odds Ratio, CI: Confidence Interval; *** *P* < 0.001; ** *P* < 0.01; * *P* < 0.05.

### 3.3. Associations with three types of excess adiposity

In multivariable adjusted logistic regression analysis, frequently having snacks, infrequent red meat consumption, avoidance of eating fatty foods and cholesterol, being male and coming from a wealthy family background was associated with BMI obesity. Fewer meals a day, frequent snack consumption, avoidance of eating fatty foods and cholesterol and being male was associated with high waist-height ratio, and frequent snack consumption and avoidance of eating fatty foods and cholesterol was associated with high waist circumference. In addition, fewer meals a day, fewer servings of vegetables a day and being male was associated with BMI overweight. Physical activity and sedentary behavior was not significantly associated with any obesity measure (see Table 3).

## 4. Discussion

The study found in a large sample of undergraduate university students across eight ASEAN countries a high prevalence of general obesity (24.2% among men and 9.3% among women). This prevalence is probably higher than in a previous study of university students from 22 developing countries that found 18.9% general overweight and 5.8% general obesity among men and 15.1% overweight and 5.2% obesity among women (with a cut off of ≥27 kg^2^ for obesity in Asian students and ≥30 kg^2^ for non-Asian students) [9]. In individual ASEAN countries such as Malaysia and Thailand a similar prevalence of general obesity was found among university students in previous studies [10]–[12],[25] compared to this study. Apart from in Laos and Myanmar, male obesity exceeded female obesity in the other countries (Indonesia, Malaysia, Philippines, Singapore Thailand and Vietnam) and overall in this university student population. The preponderance of obesity among male university students was also found among previous studies among university students in Malaysia and Thailand [10]–[12]. However, in previous national estimates of obesity (≥30 BMI) among adults over 20 years female obesity exceeded in ASEAN countries (Indonesia, Laos, Malaysia, Myanmar, Thailand and Vietnam) male obesity [2]. It is possible that emerging adult men who are attending university education are more vulnerable to transitional changes than men in the general population are and develop more likely obesity than women. Compared to first year students, higher study year studies had higher (but not significantly higher) levels of obesity in this study, as found in a recent meta-analysis of weight gain in university students [26]. This study found that students coming from a wealthier family background were more likely to have general obesity than students from poorer family backgrounds were were. This finding was also confirmed in a large sample of open-university students in Thailand [27].

The study found that frequent snacking behavior was associated with all three obesity indicators measured, as also found among university students in Pakistan [28]. The question on in between meals snacking behavior did not specify the type of snack in this study, and different definitions of snacking may influence the estimated probability of obesity [29]. In this study, making a conscious effort to avoid fat and cholesterol was associated with obesity. Other studies have also found this association [5],[9]. It is possible that in this study where intention (assessed by conscious effort) to avoid fats and cholesterol which was self-reported, did not correspond to the actual eating pattern [9]. On the other hand, students who were overweight or obese might have already adopted healthier eating behavior in order to lose weight and be more accepted by their peers [9]. Since the study design was cross-sectional, causality between dietary variables and obesity could not be established. In this study, a higher number of meals a day were protective of high WHtR, which is conforming to results from several studies [30],[31]. It is likely that eating frequency impacts obesity through a combination of different mechanisms affecting a decrease in hunger, an increase of satiety responses and a reduction binge eating [31]. Frequent (daily) red meat consumption was in this study protective from BMI obesity, while high consumption of meat was positively associated with body mass index in a national study in Finland [32] and abdominal obesity in China [33]. Although a large proportion of students (53.2%) had been having their breakfast irregularly, this study did, unlike in a previous meta-analysis of studies in Asia and Pacific regions [34], not find an association between skipping breakfast consumption and obesity.

Contrary to some previous studies [12],[28], this study did not find an association between lack of physical activity, sedentary behavior and obesity, as also found in other studies [5]. Yet, the low levels of physical activity and high levels of sedentary behavior found in this study are cause for concern, as low physical activity is a major risk factor for cardiovascular disease [35].

### Study limitations

This study had several limitations. The investigation was carried out with students from one university in each country, mostly in urban centers and inclusion of other centers could have resulted in different results. University students are not representative of young adults in general, and the obesity prevalence and its associated factors may be different in other sectors of the population. The study was a cross-sectional study and the temporal relationships between behavior practices and obesity cannot be established in such studies; further longitudinal studies are needed. Apart from anthropometric measurements, a limitation of the study was that all the other information collected was based on self-reporting. Longitudinal studies are required to investigate the obesogenic factors in this university population in ASEAN.

## 5. Conclusion

The study found a high prevalence of obesity among male and female university students across eight ASEAN countries. Specific dietary behaviors but not physical activity nor sedentary behavior were found to be associated with obesity, which can be utilized in health promotion programs addressing this university student population.
